# Supplementary material for: Flat band carrier confinement in magic-angle twisted bilayer graphene
Source: Nat Commun. 2021 Jul 7;12:4180. doi: 10.1038/s41467-021-24480-3 (PMC8263728; doi:10.1038/s41467-021-24480-3)
Supplement: Supplementary file 1 — Supplementary Information [file 41467_2021_24480_MOESM1_ESM.pdf]

Supplementary Information for ‘Flat band carrier confinement in magic-angle twisted bilayer graphene’

Nikhil Tilak[1], Xinyuan Lai[1], Shuang Wu[1], Zhenyuan Zhang[1],

Mingyu Xu[2,3], Raquel de Almeida Ribeiro[2,3], Paul C Canfield[2,3]

and Eva Y. Andrei[1*]

1 *Department of Physics and Astronomy, Rutgers, The State University of New Jersey, 136 Frelinghuysen Rd, Piscataway, NJ 08854*

2 *Ames Laboratory, U.S. Department of Energy, Ames, Iowa 50011, USA*

3 *Department of Physics and Astronomy, Iowa State University, Ames, Iowa 50011, USA.*

Corresponding author email: eandrei@physics.rutgers.edu

Supplementary Note 1: Device micrographs 2

Supplementary Note 2: Waterfall plots of dI/dV gate dependence 3

Supplementary Note 3: Coulomb diamond analysis 4

Supplementary Note 4: Additional gate dependence maps on AA and AB sites 5

Supplementary Note 5: High resolution dI/dV spatial mapping 7

Supplementary Note 6: dI/dV set-point dependence 8

Supplementary Note 7: Carrier density variation estimation in the STM device 9

Supplementary Note 8: Quantum dots in STS experiments from trivial origins 11

Supplementary Note 9: Additional Planar Tunneling Spectra at 77K 12

Supplementary Note 10: Estimation of the twist angle of PTJ device from gate dependence 12

Supplementary Note 1: Device micrographs

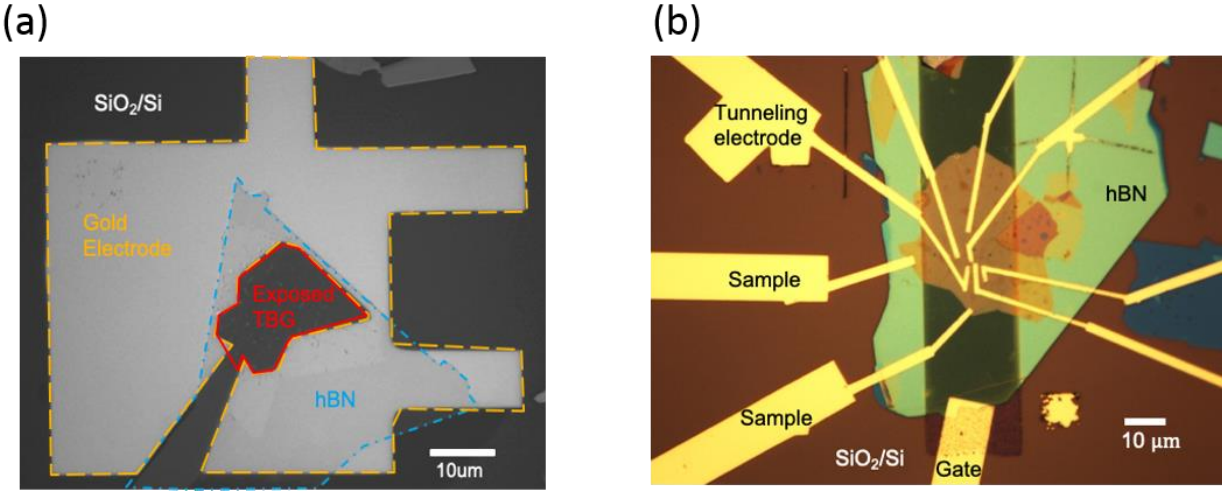

Supplementary Figure 1 Device Optical Micrographs: (a) Optical micrograph (grayscale) of the STM device. The Gold electrode (yellow dashed lines), TBG region (red solid line) and hBN (cyan dashed line) are marked. (b) Optical micrograph of the planar tunneling device.

Supplementary Note 2: Waterfall plots of dI/dV gate dependence

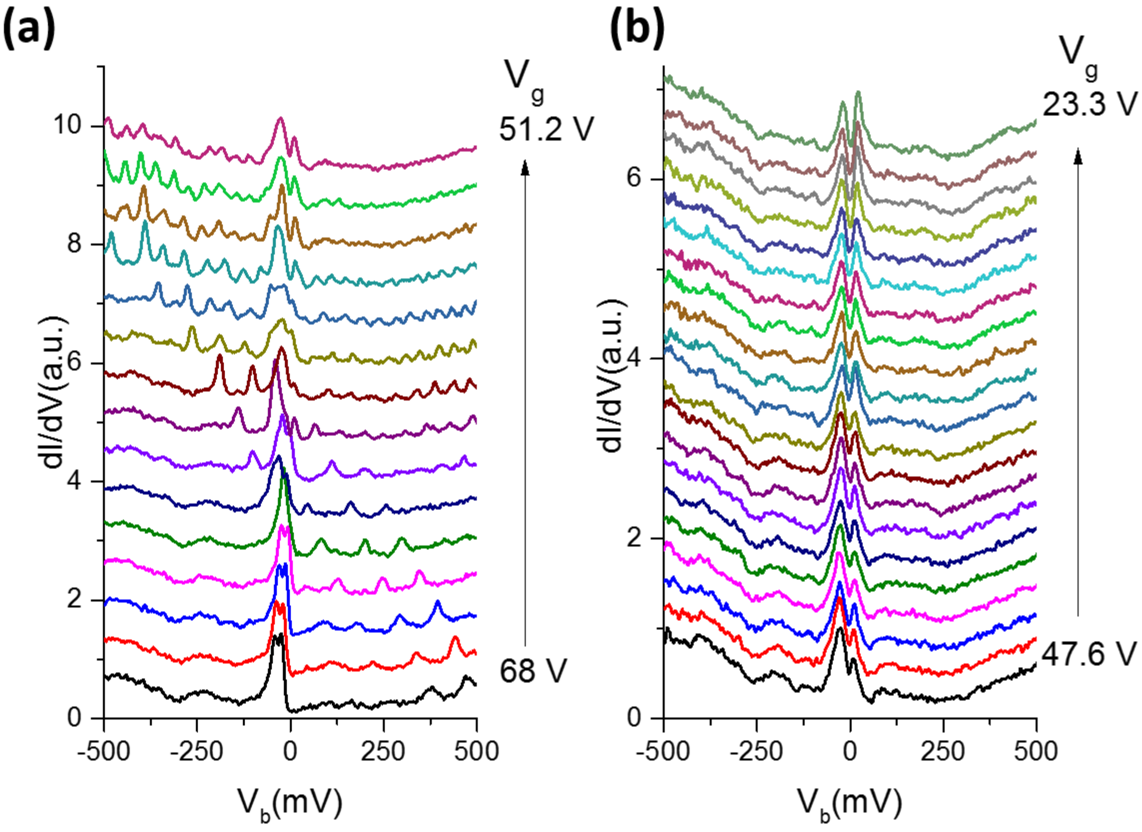

Supplementary Figure 2 line plots of dI/dV gate dependence in STM device: dI/dV evolution with gate voltage from 68 V to 51.2 V (a) and from 47.6 V to 23.3 V (b) respectively (same data as main Fig 2). Quantum dot peaks are no longer visible in the bias range when the gate voltage is below about 50 V.

Supplementary Note 3: Coulomb diamond analysis

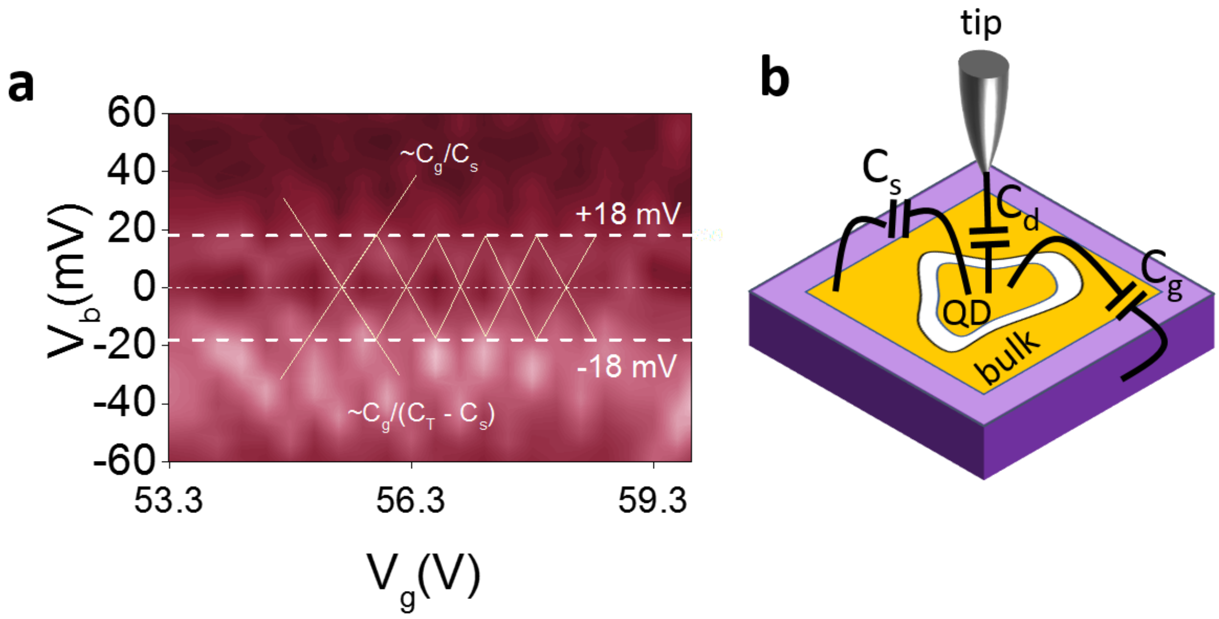

Supplementary Figure 3 Coulomb diamond analysis: (a) A magnified view of the $dI/dV_b(V_b, V_g)$ map from Main Fig. 2a which shows the Coulomb diamonds near the low bias range. The yellow lines are guides for the eyes. The slopes of the coulomb diamond have also been labelled. C_g, C_s and C_d are the capacitances between gate-dot, bulk-dot and tip-dot respectively as illustrated in panel (b). The total capacitance $C_T = C_g + C_s + C_d$.

Each quantum dot is capacitively coupled to the backgate, the tip and the nearby conducting regions of the sample. Tunneling from the tip into the dot and then from the dot to the nearby conducting regions of the sample requires a precise alignment of energy levels of the dot with those of the tip and the sample (Main Fig. 3d). This alignment depends on all three capacitances, the sample bias and the gate voltage as discussed below.

First let's consider the case where the bias voltage is smaller than the potential barrier surrounding the quantum dot. In this case the only way in which electrons can tunnel from the tip into the bulk of the sample is by first tunneling into the quantum dot. As the backgate voltage is increased (decreased), the energy states in the quantum dot are lowered (raised). When an energy level of the quantum dot aligns with the sample Fermi level, electrons can tunnel into the quantum dot from the tip and out of the quantum dot into the bulk of the sample. This is observed as a sharp increase in the tunneling current. Because of Coulomb repulsion, the next electron needs an additional energy (E_{add}) to tunnel into the dot. The additional energy is, in general, the sum of the charging energy (E_C) and the orbital spacing (Δ). The orbitals have some degeneracy. The energy Δ is only needed once all the states in a particular orbital have been filled and the electron is added to the next orbital. The charging energy depends on the total capacitance of the quantum dot ($C_T = C_g + C_s + C_d$) via $E_C = e^2/C_T$. The orbital spacing Δ depends on the size of the dot and the details of the confining potential. The next higher energy state becomes accessible when the gate voltage is further increased by a value $\Delta V_g = e/C_g$.

At larger sample bias (V_b), the tip can also gate the confined states in the dot. A negative (positive) sample-bias is equivalent to a positive (negative) tip-bias and has the same effect as a positive (negative) backgate voltage. The same quantum dot state can be aligned with the tip/sample Fermi level for a continuous set of linear combinations of sample bias and backgate voltage. This is the origin of the series of oblique lines seen in the gate dependence maps (Main Fig. 2). The slope of these lines contains information about the capacitances between the tip-dot (C_d)

and the dot-sample ($\mathrm{C_s}$). To further study the role played by the tip-dot capacitance we recorded the tunneling set-point dependence of the spectra (see Supplementary Figure 7).

By analyzing the Coulomb diamonds as outlined in Supplementary Figure 3a, we measured the charging energy of our quantum dot, $\mathrm{E_C}$ = (18 ± 1.4) meV, which gives $\mathrm{C_T}$ = (8.8 ± 0.7) aF. The capacitance between the dot and the back gate in our sample is $\mathrm{C_g}$ = 2.4 ± 0.6 aF. Modelling the quantum dot as a circular metallic disc, we estimate the size of the quantum dot as 61 ± 4.8 nm. This matches reasonably well with the size (~50-60 nm) of the conducting region where the spectrum in Fig. 2a was collected. It is worth noting that the apparent size of the quantum dot as well as the height of the tunneling barrier depend on the applied gate voltage. Approximating the quantum dot as a flat disc with diameter 60 nm and using a carrier effective mass of $\mathrm{m^*} = 0.05\mathrm{m_e}$ from Bernal Bilayer Graphene[1], we found that $\Delta \approx 2\ \mathrm{meV}$ which is negligible compared to the measured charging energy of 18 meV. Δ can therefore safely be neglected.

By analyzing the slopes of the charging lines we calculated the ratio of the three capacitances as $\mathrm{C_g} : \mathrm{C_s} : \mathrm{C_d} :: 1 : 37 : 36$. This implies that although $\mathrm{V_b}$ is typically small compared to $\mathrm{V_g}$, the tip is very efficient at gating because of its proximity to the dot. This explains the sign of the slope of the oblique charging lines in the gate dependence map (Main Fig. 2a).

Supplementary Note 4: Additional gate dependence maps on AA and AB sites

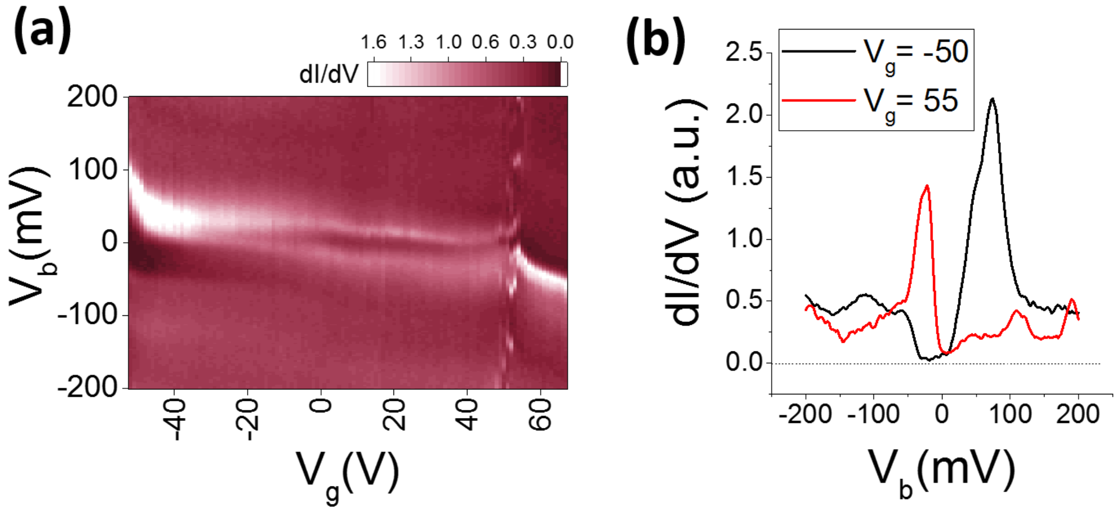

Supplementary Figure 4 Full-range gate dependence maps: (a) A wide gate-range $dI/dV_b(V_b,\ V_g)$ map at an AA site located in the same region as Main Fig. 3a. It takes about 80 V of gate voltage to go from fully filled bans to fully empty bands based on the capacitance between the backgate and sample. (b) Individual dI/dV_b spectra when the bands are fully filled ($V_g = 55\ V$) and fully empty ($V_g = -50\ V$). The superlattice gaps can be seen near the fermi level in both curves but the gap on the other side of the flat bands is not clearly visible because of a large background signal at higher biases.

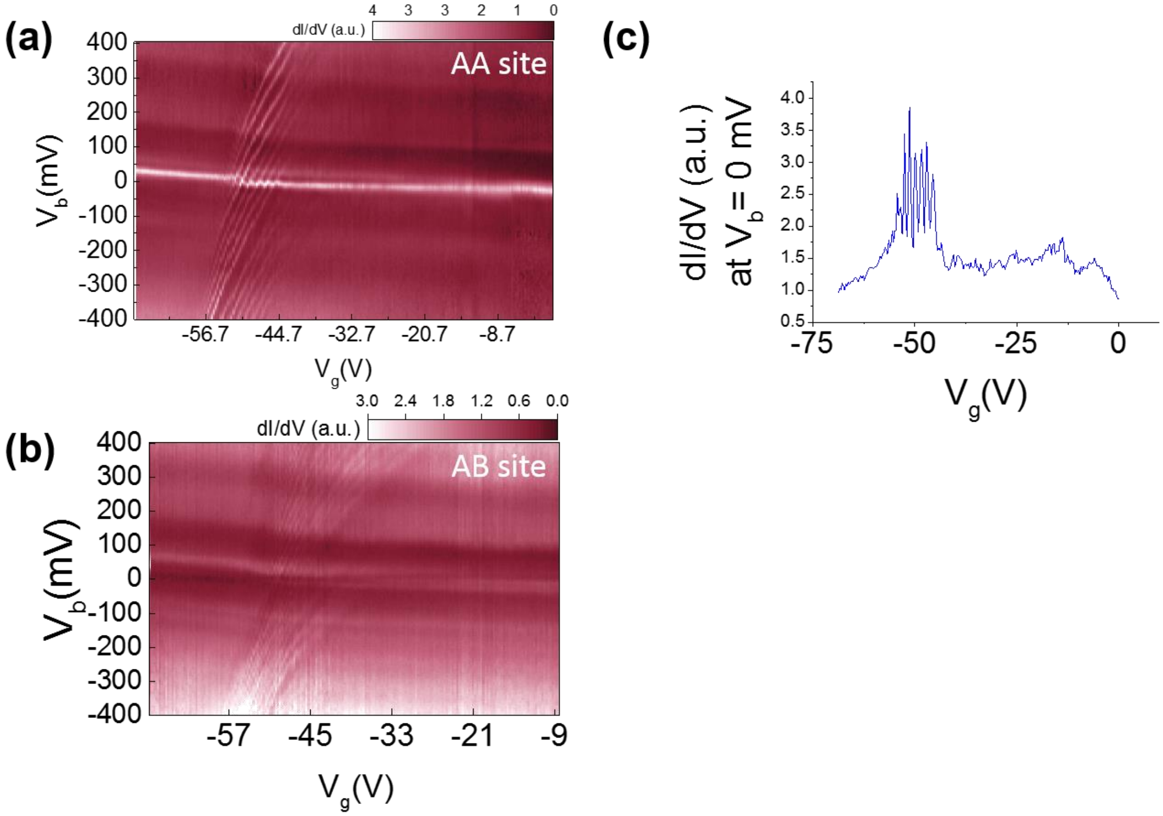

Supplementary Figure 5 Signatures of confinement in zero-bias conductance: Charging peaks seen on the bottom edge of the hole side flat band in a different region in the sample measured at an AA site (a) and at an AB/BA site (b). The zero bias conductance measured from the data in (a) is shown in (c). The single electron charging events at zero bias appear as sharp peaks.

Although the results in the main figures demonstrate confinement near the electron-side band-edge, there is nothing special about the electron-side band. Confinement can also exist in the hole side band edge. We measured confinement states near the hole side band edge at a different position in the sample. results are shown in Supplementary Figure 5b.

Supplementary Note 5: High resolution dI/dV spatial mapping

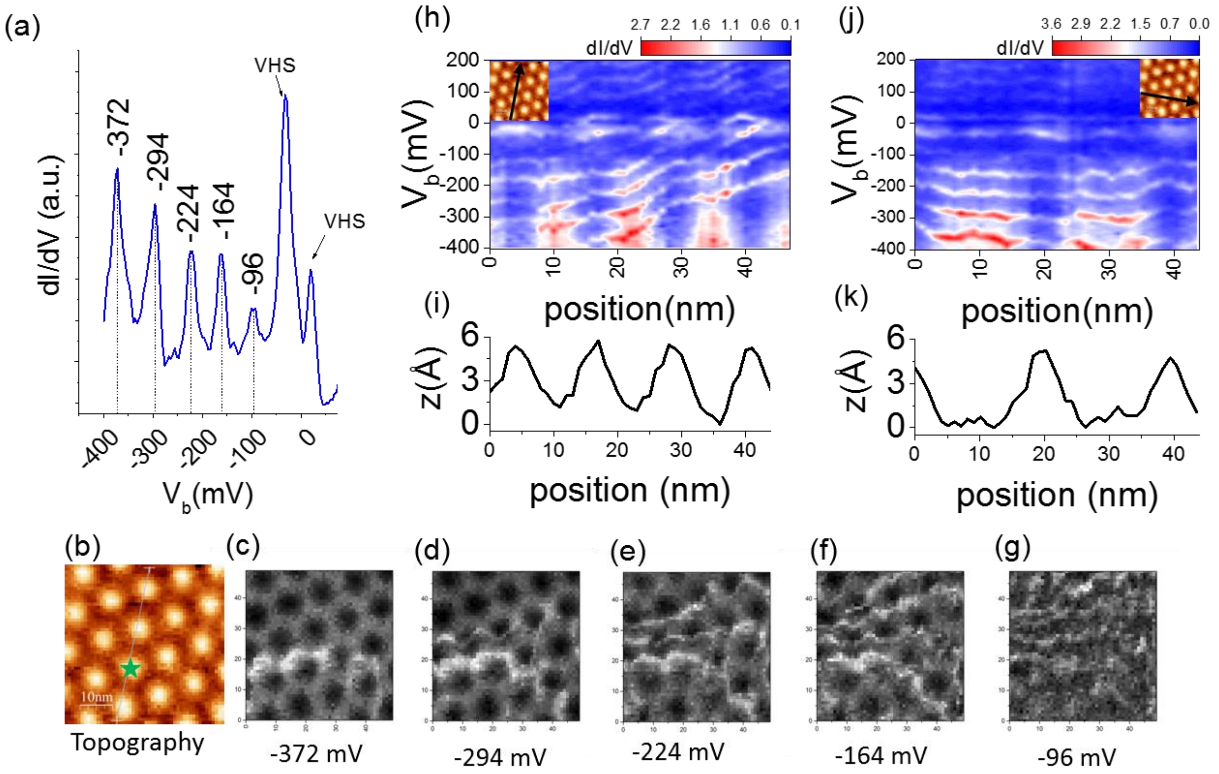

Supplementary Figure 6 dI/dV spatial maps at V_g = 57 V: (a) shows an STS spectrum measured at position marked by the green star in topography (b). Charging peaks corresponding to the Quantum dot states are seen at -372 mV, -294 mV, -224 mV, -164 mV and -96 mV respectively. The dI/dV maps corresponding to these energies are shown in figures (c)-(g). Bright wavefronts corresponding to each QD state are seen in these maps. Figure (h) maps the position dependence of dI/dV at various points along the direction labelled by the arrow in the inset. Figure (i) shows the corresponding height variation along the same direction. The Quantum dot peaks in (h) show a general trend of shifting higher in energy (less negative) as the tip moves outwards towards the edge of the Quantum dot. This is an indication that the tip-dot capacitance reduced as the tip moves towards the edge of the Quantum dot. This reduces the tip-gating efficiency and thus the same QD state appears at higher bias voltages near the edge of the dot. The change in energy briefly slows down when the tip is scanning an AA site as marked by the dotted lines. This can be attributed again to the decrease in the gating efficiency of the tip as the tip-dot distance increases in the z direction while scanning the AA site. Figures (j) and (k) are similar to (h) and (i) except the direction is as indicated by the inset which is perpendicular to the direction for (h) and (i). Along this direction the Quantum dot peaks appear to have nearly constant energy. This is to be expected since this direction is along the QD wavefronts as seen in the dI/dV maps.

To further study the Quantum dot at a smaller scale we performed dI/dV mapping in the region marked by the magenta square in Main Fig. 3a at a gate voltage of 57 V. Supplementary Figure 6a shows the dI/dV spectrum measured at the position labelled by the green star in Supplementary Figure 6b. In addition to the flat bands on either side of 0 mV bias, several peaks corresponding to the charging of QD states are visible in the spectrum. In Supplementary Figure 6(c-g) the dI/dV maps at these energies are shown. In the maps the charging states are seen as bright wavefronts. At higher energies all the wavefronts with lower energies are also visible. As the energy is increased from negative values towards 0, the wavefronts appear to move outward toward the edge of the QD. This can be explained by considering the decrease in the tip-dot capacitance as the tip moves from the center of the dot towards the edge. As the tip-dot capacitance decreases, the tip gating efficiency goes down. This means that we need a larger (less negative) bias in order to reach the same QD energy level.

This effect can be seen more directly by mapping the position dependence of the QD energy levels along a direction which points towards the edge of the QD (Supplementary Figure 6h) and a direction orthogonal to it (Supplementary Figure 6j). In Supplementary Figure 6h, the QD states can be seen moving up in energy as the tip moves outward towards the edge of the dot. Interestingly the increase in the QD peak energies seems to slow down when the tip is close to the AA regions. This again can be explained by considering the reduction in the tip-gating efficiency as the tip-sample height is greater by about 0.5 nm (Supplementary Figure 6i), when the tip is near the top of the AA sites owing to the larger LDOS there.

In contrast, in Supplementary Figure 6j, the energy of the QD states doesn't seem to change much. This is because the tip-dot capacitance doesn't change appreciably when the tip moves along this direction. Notice that this direction is roughly tangential to the wavefronts.

Supplementary Note 6: dI/dV set-point dependence

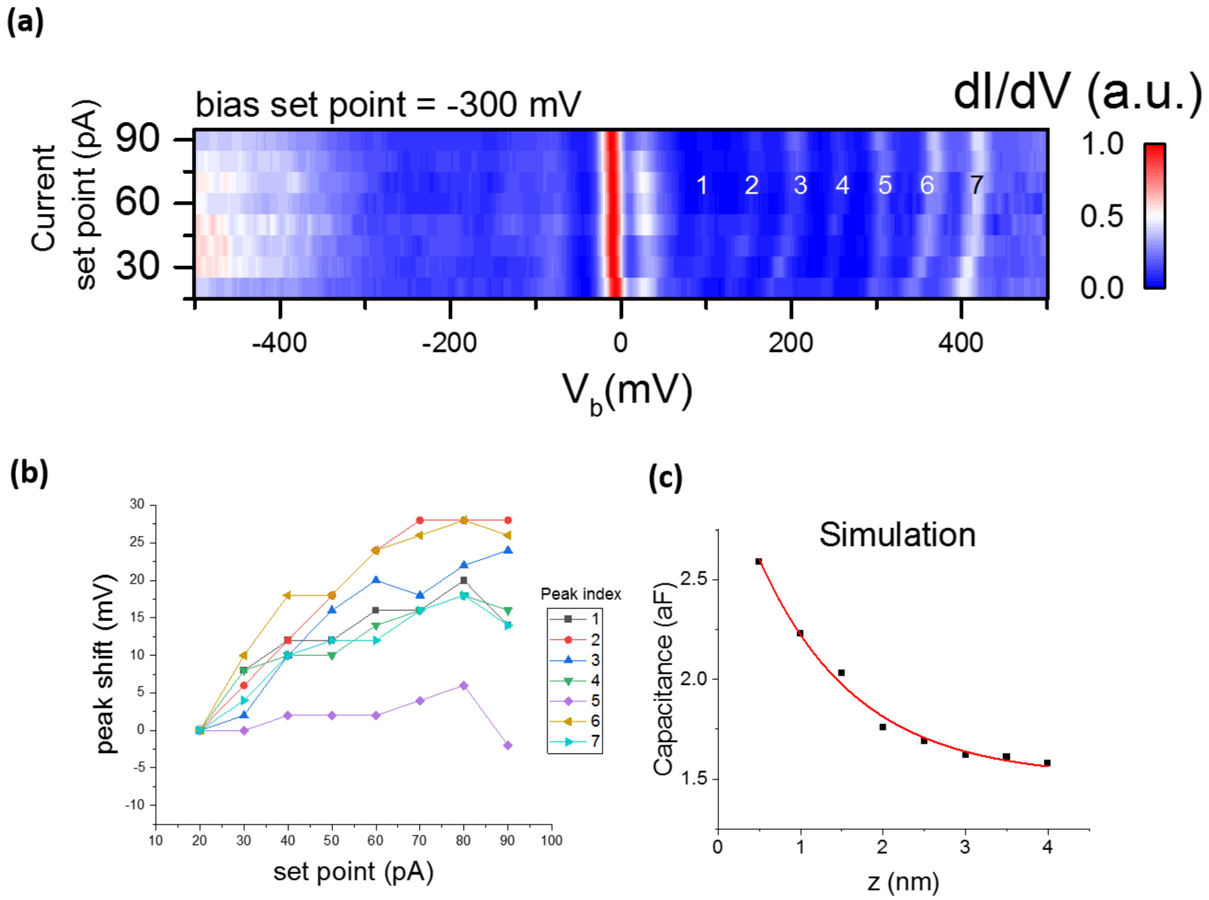

Supplementary Figure 7 Set point dependence of the confinement state energies: (a) is a set point dependence dI/dV map. current set points were varied between 20 pA and 90 pA while maintaining an initial bias set-point of -300 mV. The bright lines at positive biases represent confined states labelled by numbers 1 -7. As the set point in increased the energies of the states systematically increase. (b) plots the shift in the energy of each of the 7 states as the set point is increased from 20 to 90 pA. The states shift upwards in energy by about 20 meV. Interestingly state 5 doesn't change significantly in energy. The capacitance between the tip and the sample should increase as the tip is brought closer to the sample. We simulated our tip as a sphere and the sample as a circular disc. The calculated Capacitance between the tip and the sample is plotted in (c) as a function of tip-sample distance. The capacitance indeed increases non linearly as the tip-sample distance is reduced.

Another way of changing the tip-dot capacitance is by changing the initial set point at which the dI/dV spectrum is measured. To this end, we positioned the tip on top of an AA site and measured the dI/dV spectra at initial current set points of 20 pA to 90 pA in increments of 10 pA keeping the initial bias set point constant at -300 mV. In Supplementary Figure 5a we plot the spectra as a map. The bright lines, most clearly seen at positive bias, are QD states. They have been labelled 1-7. Observe that as the set point is increased, the states move to higher energies. This increase in energy is plotted in Supplementary Fig. 5b. Almost all the states increase in energy by about 20 meV. Interestingly state 5 doesn't seem to move much. We have been unable to explain this result.

As a sanity check we simulated the system as a spherical tip with radius 10 nm and the Quantum dot as a circular disc of radius 100 nm and calculated the capacitance between them for different tip-sample distances. The results are shown in Supplementary Figure 5c. Each data point represents a simulation performed with a different tip-sample distance. The capacitance increases non-linearly as the tip-sample distance is reduced.

Supplementary Note 7: Carrier density variation estimation in the STM device

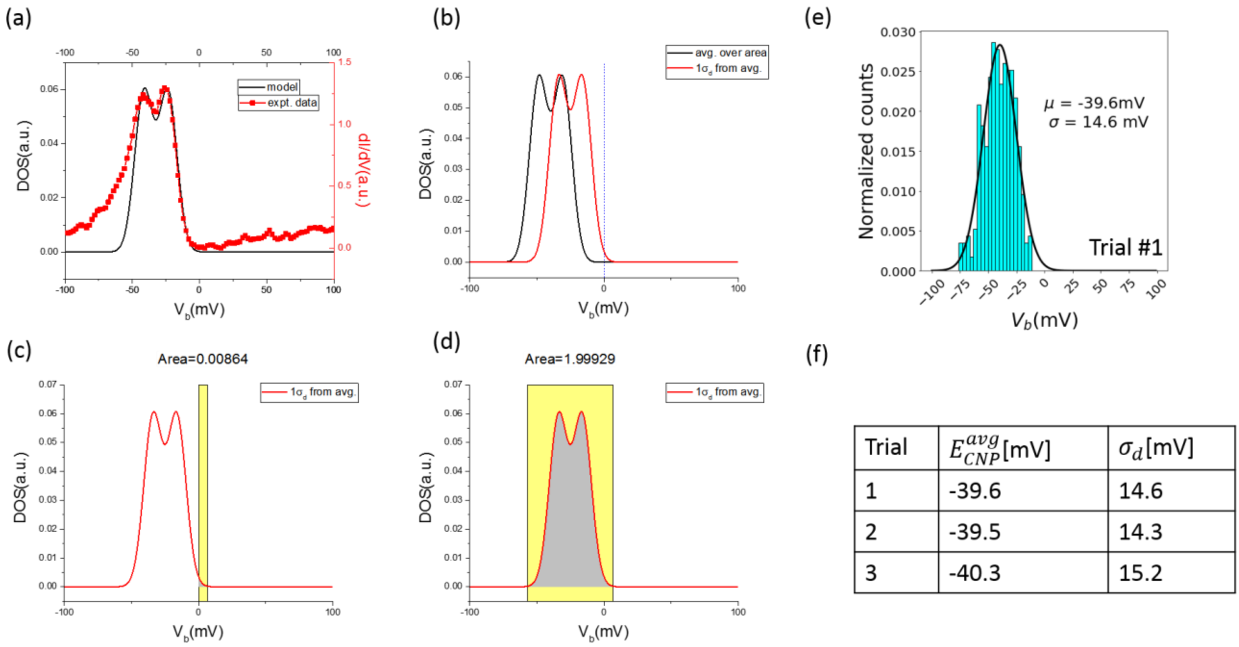

Trial	E_{CNP}^{avg}[mV]	σ_d[mV]
1	-39.6	14.6
2	-39.5	14.3
3	-40.3	15.2

Supplementary Figure 8 Carrier disorder estimation: (a) The flat bands are modelled as two gaussians with centers separated by 18 mV and FWHM of 16 mV each. The model (black curve) is superimposed on an experimental dI/dV curve (red) to show the goodness of fit. The model underestimates the density of states at the lower band edge, most likely because of a non-linear background signal. (b) The model is plotted according to the average CNP position in the map (black) and 1 std. dev. shifted to the right (red). (c) the gray shaded area under the DOS curve integrated from the fermi level (0 mV) to the upper band edge (6.9 mV) represents empty states. (d) the gray shaded area under the DOS curve integrated from the lower band edge (-57.1 mV) to the upper band edge (6.9 mV) represents the total states within the flat band. (e) An example of the charge neutrality point variation in the dI/dV spatial map (main Fig 3) displayed as a histogram. 360 points were randomly sampled from the dI/dV map and the CNP energy at those points was noted. The black line is a gaussian(μ, σ) curve fitted to the histogram. (f) shows the tabulated results for 3 such trials.

In previous STM experiments on Monolayer Graphene (MLG) supported by a SiO_2, hBN or MoS_2 substrate, the carrier density variation was estimated by measuring the average variation of the Dirac point energy (E_d) and translating that to a carrier density variation using the well-known formula for the $E_d(n)$ for MLG: $E_d^2 = \hbar^2 v_F^2 \pi\, n$.

In TBG, the exact analytical expression for the dispersion is not known. Therefore, the simple approach used for MLG cannot be directly used in the case of TBG. In what follows, we attempt to get an estimate for the carrier density disorder in our sample.

First, to quantify the variation of the charge neutrality point (E_{CNP}) in the sample we used the following procedure-

1. A large dI/dV spatial mapping was acquired at a gate voltage $V_{g0} = 68\ V$ (Main Fig 3a) and the energy of the charge neutrality point (E_{CNP}) in the dI/dV spectra taken at various positions in the mapping was recorded. This was done with the help of a Python script which randomly samples 360 points in the 3600 point dI/dV map. Then the STS at each of these points is extracted from the data. The dip in the dI/dV spectrum between the two VHS is manually assigned as the CNP. The actual CNP position may not be exactly at the mid-point between the VHSs but it is a reasonable approximation for the purposes of this calculation.
2. The charge neutrality point energy distribution is plotted as a histogram and a gaussian was fitted to it. The standard deviation (σ_d) of the fitted gaussian is taken as a measure of the chemical potential variation in the sample.
3. This procedure of randomly sampling 10% of the points in the grid was repeated 3 times in order to get a better sense of the average variation.
4. The average σ_d using this procedure is $\sigma_d = (14.7 \pm 0.5)$ mV and the average position of the CNP was $E_{CNP}^{avg} = -39.8 \pm 0.4$ mV. The error bars represent one standard deviation calculated from the 3 trials.

After this procedure we estimated the carrier density disorder (n_d) as follows-

1. The separation of the VHS and the FWHM were recorded for many points from the STS map. This yielded an average VHS separation of 18 mV and the FWHM of each band as $w = 16\ mV$.
2. The DOS of the flat bands was modelled as the sum of two gaussians with the centers separated by 18 mV and with FWHM $w = 16$ mV. The height of the gaussians was determined by the area under the curve which was arbitrarily set to 1. The exact value of the area does not affect the following calculations.
3. The band edges are taken as $E_{CNP}^{avg} \pm 2w$. For the measured average value $E_{CNP}^{avg} = -39.8$ mV average position the band edges are located at (-71.8 mV, -7.8 mV).
4. This implies that the $E_F = 0\ mV$ is located inside the gap between the upper flat band and the upper remote band i.e. the flat bands are fully filled. Now consider a spatial position in the sample where the CNP position is shifted with respect to the average value by 1 σ_d due to doping disorder. The upper band edge will then be shifted to $\delta = E_{CNP}^{avg} + 2w + \sigma_d$ mV. The measured value of $\sigma_d = 14.7\ mV$ places the band edge at 6.9 ± 0.9 mV where the error bar represents the uncertainty in the values of σ_d and E_{CNP}^{avg}.
5. The states from the E_F to the band edge, i.e., 0 mV to 6.9$\pm$0.9 mV are empty. Integrating the simulated DOS in this range gives an area of $\int_0^{6.9\pm0.9} DOS(E)dE = 0.009 \pm 0.003$ units. Comparing this to the integration of the entire bands $\int_{-57.1}^{6.9} DOS(E)dE = 1.999$ gives an empty band fraction of $\frac{0.009\pm0.003}{1.999} = 0.0045 \pm 0.0015$. Since the flat bands at the measured twist angle of 1.12 deg can accommodate $2n_s = 5.82 \times 10^{12}/cm^2$ carriers, a 0.0045$\pm$0.0015 fraction of that represents

$(2.62 \pm 0.87) \times 10^{10}/cm^2$ carriers. This gives an estimate of the carrier density variation in the sample.

Supplementary Note 8: Quantum dots in STS experiments from trivial origins

There are various scenarios where resonant states can appear in STS. One trivial possibility is that a weakly conducting impurity can be attached to the STM tip near the tunneling junction. This impurity can act like a Quantum dot and produce sharp peaks in STS whenever the sample or tip Fermi level aligns with its confinement states. Since such a Quantum dot is always surrounded by a tunneling barrier which is independent of the gate voltage, these peaks show up at all gate voltages. Main Figure 2c shows dI/dV spectra gathered at different gate voltages. It can be seen that the resonant states exist only for a small range of gate voltage. Furthermore, we reformed the tip apex by applying bias pulses (3-6 VDC for 200 ms) while scanning the gold electrode. This procedure re-sculpts the very apex of the tip near the tunneling junction and is almost equivalent to changing tips. This procedure was repeated until sharp topographic scans were obtained. Then dI/dV spectra were measured on the Gold electrode to ensure that the spectrum was flat in the energy range of interest ($\pm$ 500 mV). The resonant peaks in the TBG region were found to be reproducible before and after such tip cleaning. Therefore, we can safely rule out the possibility of a nanoparticle loosely stuck to the tip as a source of these peaks.

Another possibility is that the tip is invasive and creates local p-n junctions[2] or causes band bending locally[3,4] in order to produce confinement. We can safely eliminate this possibility because the Quantum dot states occur in specific locations in our sample unlike tip-induced states which should not be position dependent.

Supplementary Note 9: Additional Planar Tunneling Spectra at 77K

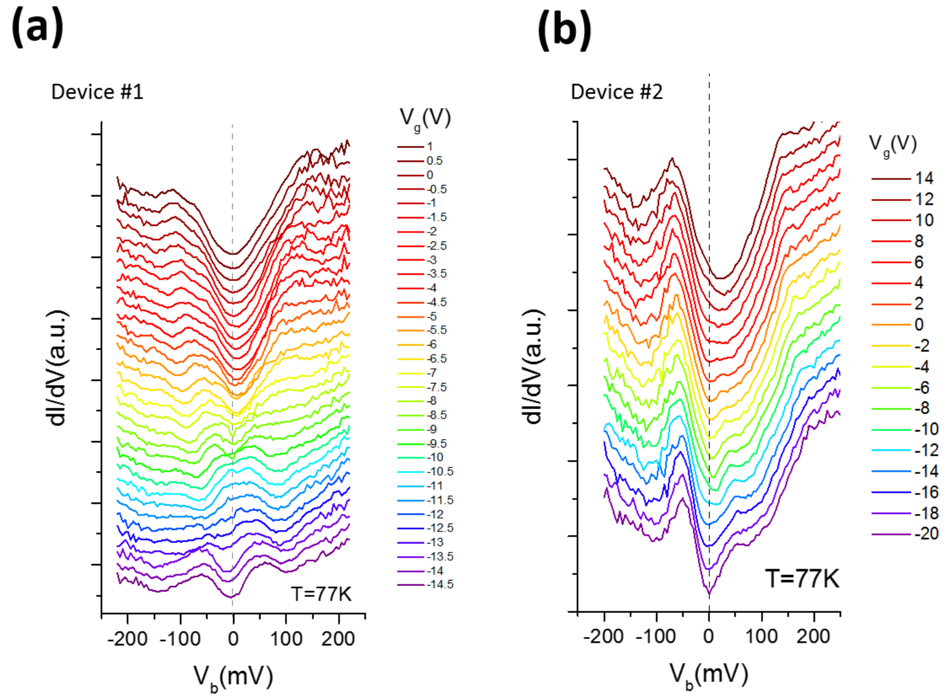

Supplementary Figure 9 additional dI/dV gate dependence from planar tunneling device: dI/dV (V_b, V_g) of device #1, #2 at 77K. Device #2 shows a similar doping effect as device #1 which was presented in the main paper, confirming the reproducibility of our results.

Supplementary Note 10: Estimation of the twist angle of PTJ device from gate dependence

As a check we also estimate the twist angle from the gate voltage needed to completely fill the flat bands using equations (2) and (3) as described in the main paper. At $\mathrm{V_g} = 0$, the average of the energies of the upper and lower VHS approximately corresponds to the energy of the CNP of the sample (~-80 mV). This indicates that the sample is electron-doped, presumably due to the work function difference between the tunneling electrode and the TBG. Extrapolating the CNP position to the Fermi level ($\mathrm{V_b} = 0$) indicates that the sample is charge neutral at a $\mathrm{V_g} = -9.7\ \mathrm{V}$ labeled by the dashed white line in Fig 4c. Since our gating ability is limited to $\pm 14.5\ V$ by the breakdown voltage of the bottom hBN dielectric layer we were unable to completely empty the flat bands. Nonetheless, since the flat bands start emptying at $\mathrm{V_g} \approx -(2 \pm 1)\ \mathrm{V}$ and are filled to the CNP at $\mathrm{V_g} \approx -9.7\ \mathrm{V}$ we can estimate that it takes about $15.4 \pm 2\ \mathrm{V}$ of gate voltage to completely empty the bands. Inserting $(\mathrm{V_g} - \mathrm{V_{g0}}) = 15.4\ \mathrm{V}$, $d_1 = 70\ \mathrm{nm}$ (thickness of bottom hBN) and $d_2 = 0$ in equation (3) yields a charge density of $(4.9 \pm 0.6) \times 10^{12}\mathrm{cm}^{-2}$. Since this charge density corresponds to $2n_S$, equation (2) gives a twist angle of $\sim(1.0 \pm 0.1)^{\circ}$.

Supplementary References

1 Li, J. *et al.* Effective mass in bilayer graphene at low carrier densities: The role of potential disorder and electron-electron interaction. *Physical Review B* **94**, 161406, doi:10.1103/PhysRevB.94.161406 (2016).

2 Jiang, Y. *et al.* Tuning a circular p–n junction in graphene from quantum confinement to optical guiding. *Nature Nanotechnology* **12**, 1045-1049, doi:10.1038/nnano.2017.181 (2017).

3 Dombrowski, R., Steinebach, C., Wittneven, C., Morgenstern, M. & Wiesendanger, R. Tip-induced band bending by scanning tunneling spectroscopy of the states of the tip-induced quantum dot on InAs(110). *Physical Review B* **59**, 8043-8048, doi:10.1103/PhysRevB.59.8043 (1999).

4 Li, S.-Y. *et al.* Nanoscale detection of valley-dependent spin splitting around atomic defects of graphene. *2D Materials* **6**, 031005, doi:10.1088/2053-1583/ab2074 (2019).
